# L-serine: Neurological Implications and Therapeutic Potential

**DOI:** 10.3390/biomedicines11082117

**Published:** 2023-07-27

**Authors:** Soe Maung Maung Phone Myint, Liou Y. Sun

**Affiliations:** Department of Biology, University of Alabama at Birmingham, Birmingham, AL 35233, USA; sphonemy@uab.edu

**Keywords:** L-serine, Alzheimer’s disease, neuroinflammation, metabolism, D-serine, Parkinson’s disease, multiple sclerosis, brain injury

## Abstract

L-serine is a non-essential amino acid that plays a vital role in protein synthesis, cell proliferation, development, and sphingolipid formation in the central nervous system. It exerts its effects through the activation of glycine receptors and upregulation of PPAR-γ, resulting in neurotransmitter synthesis, neuroprotection, and anti-inflammatory effects. L-serine shows potential as a protective agent in various neurological diseases and neurodegenerative disorders. Deficiency of L-serine and its downstream products has been linked to severe neurological deficits. Despite its crucial role, there is limited understanding of its mechanistic production and impact on glial and neuronal cells. Most of the focus has been on D-serine, the downstream product of L-serine, which has been implicated in a wide range of neurological diseases. However, L-serine is approved by FDA for supplemental use, while D-serine is not. Hence, it is imperative that we investigate the wider effects of L-serine, particularly in relation to the pathogenesis of several neurological deficits that, in turn, lead to diseases. This review aims to explore current knowledge surrounding L-serine and its potential as a treatment for various neurological diseases and neurodegenerative disorders.

## 1. Serines and Their Role in the Central Nervous System

Despite being categorized as a non-essential amino acid, L-serine has repeatedly demonstrated beneficial effects by acting as a precursor to crucial molecules required for protein synthesis, cell proliferation, and development. Its role in the formation of sphingolipids in the central nervous system is essential for neural differentiation and survival [1,2]. L-serine is produced via the phosphorylated pathway in the mammalian brain and is derived from glucose from glycolysis rather than phosphenolpyruvate from gluconeogenesis [3]. However, L-serine uptake from blood across the blood-brain barrier is inefficient when compared to other essential amino acids [4].

L-serine also serves as a precursor to glycine and D-serine, which are important for excitatory glutamatergic neurotransmission. D-serine is involved in long-term potentiation and synaptic plasticity, while glycine acts on extra-synaptic NMDARs [5,6]. L-serine is produced primarily in glial cells, specifically astrocytes, and is converted to D-serine via the enzyme serine racemase [7].

While D-serine can cause NMDA-mediated excitotoxicity, L-serine has been found to exert neuroprotective effects by mitigating neurotoxicity through the activation of the glycine receptor and increasing the expression of PPAR-y, which plays a crucial role in macrophage and microglia polarization [8,9,10,11,12]. Following a neurological disease, glial cells are activated and contribute to the inflammatory response [13,14]. Microglial cells can shift to certain phenotypes, with the M1 phenotype producing reactive oxygen species that can initiate cell death while the M2 phenotype allows for repair through phagocytosis and anti-inflammatory factor production [15,16].

L-serine plays a role in the polarization of macrophages to certain phenotypes and has anti-inflammatory effects through the downregulation of microglia and astrocyte proliferation and activation [10,17]. It reduces the production of proinflammatory cytokines and serves as a protective agent, particularly after injury. A recent study has suggested that neuroinflammation that appears as a result of traumatic brain injury at high altitude is ameliorated by L-serine, likely due to the mechanism of microglial polarization reducing neuroinflammation in the process [18]. All in all, L-serine in addition to serving as a protective agent, can even reverse the increase in volume of brain lesions and contributes to the healing process of brain tissue [18].

## 2. Serines and Their Role in Metabolism

**Figure 1:**
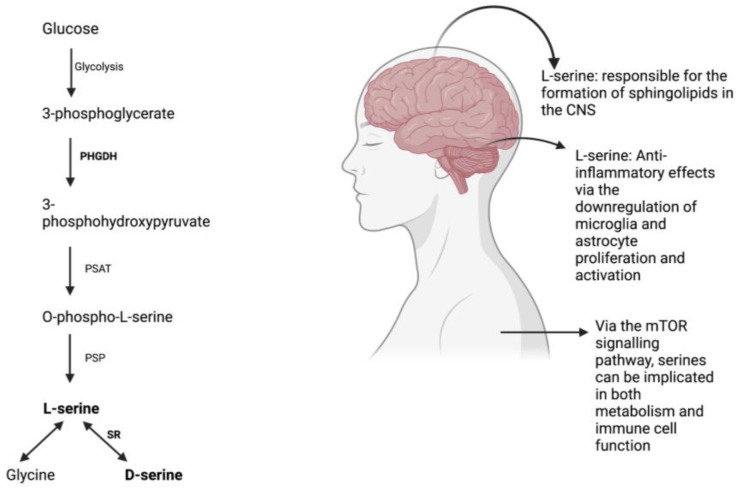
L-serine synthesis via the phosphorylated pathway [67] and its effects on the body.

The acquisition of serine by our cells follow along four major pathways: through the extracellular environment, from cellular proteins that have degraded, from glycine transamination and from glucose and glutamate *de novo* [19]. As previously mentioned, serines are crucial in the growth and proliferation of our cells, produced mainly by the de novo synthesis of serine [20]. An important molecule that contributes and coordinates the way in which nutrients and growth factors are available for cells to grow, divide and differentiate is mammalian target of rapamycin (mTOR), a serine-threonine kinase [21]. mTOR upregulates certain key enzymes to promote the synthesis and metabolism of serine [22] and partakes in functions that support innate and adaptive immune responses [23]. mTOR enhances glycolysis and enable the differentiation, proliferation of effector T cells [24] and mTORC1 exhibits increased activity during the pro- and pre-B stages during the developmental stages of mouse B cells [21]. In terms of innate immunity, serine metabolism through mTOR contributes directly to the growth and proliferation of many innate immune cell. Specifically, mTORC1 appears to be activated and is crucial when producing IFN-γ, a key cytokine produced by NK cells [25]. Research has shown how the restriction of rate of glycolysis tends to directly also restricts the production of IFN-γ [25]. Additionally, in neutrophils mTOR is stimulated by IL-23 and the inhibition of the mTOR pathway tends to have effects that in turn reduces the production of IL-17 and IL-22 induced by IL-23 [26]. Taken altogether, we can see how serine is implicated in metabolism, particularly in relation to the immune system through the mTOR signaling pathway. Serines and the role they play in the central nervous system, metabolism, and the production of serines via the phosphorylated pathway are depicted in Figure 1.

## 3. Serines and Parkinson’s Disease (PD)

L-serine because of its neuroprotective properties have been utilized in a myriad of ways to treat a vast array of neurological diseases ranging from epilepsy to Alzheimer’s Disease (AD) [27,28]. L-serine treatment has been shown to improve behavior, EEG and seizure frequency in the variants that no longer possess or overexpress the NMDAR [29]. One of the earliest studies (1993) that provided a comparison of the levels of D-serine in the human temporal cortex in the normal, Alzheimer and Parkinson’s models have shown to have no significant differences [30]. However, subsequent studies have shown the efficacy of using D-serine to target Parkinson’s disease and therefore implicating the role D-serine plays in Parkinson’s. A study in 2011 showed that D-serine and Serine Racemase are both found in the astrocytes and tyrosine hydroxylase positive neurons of the substantia nigra [31]. Because of the indication that D-Ser and SR are localized in those specific areas, it is surmised that D-serine might also be present in the Parkinson’s Disease implicated areas and hence by extension causing them to believe that the levels of D-ser and SR maybe somewhat affected during the development of Parkinson’s in the mouse model (MPTP/p) [31]. In the study after the induction of PD through the administration of MPTP for 5 weeks, despite the number of TH+ neurons being reduced dramatically, the levels of D-ser and SR were lower and not observed in the TH+ neurons of the substantia nigra but the levels of D-Ser and SR were higher in both astrocytes and nondopaminergic neurons in the striatum [31].

Another study in 2019 also found that D-serine levels were lower in both the substantia nigra and the cerebrospinal fluid of L-DOPA free PD patients [32]. MPTP treatment increased the levels of both D-aspartate and D-serine in the putamen of the monkey. Normalization of the levels of D-serine in the substantia nigra occurred after dopaminergic denervation [32]. This suggests that there could be a possible connection between the glutamatergic and dopaminergic neurotransmission in PD and that D-serine might be implicated due to their involvement in NMDAR transmission [32]. The 2019 study is more so an extension of the 2012 one that has suggested of the possibility of a D-serine adjuvant ameliorating behavioral and motor symptoms in PD [32].

A study performed on a PD rat model found that there were disturbances in the levels of glutamate transporters in the striatum even though they attributed the enhancement in levels of D-serine content to the pathophysiology of PD [33]. The disturbances in D-serine and henceforth the alteration in the levels of D-serine were noted in the study, however [33]. By proxy of D-serine being a product of L-serine via serine racemase, L-serine could be implicated/used in the treatment of Parkinson’s disease. As such, there is great evidence pointing to the participation of the L-serine and D-serine in the pathogenesis of PD although there are contradictory assessments of the levels of these specific amino acids in the regions of the brain of PD patients.

## 4. Serines and AD

A 1995 study on the concentration of free D-serine in normal and Alzheimer’s human brain suggested that there are no significant differences between their levels [34]. However, because D-serine serves as an NMDAR agonist and the implication of NMDAR in the pathogenesis of AD, it might be counter intuitive to exclude D-serine at all. Studies have suggested that drugs used to target the function of NMDARs were able to restore some function of the subsequent downstream cascades and therefore may help in alleviating AD [35]. D-serine levels in AD were further studied in 2015 which showed increases in level of D-serine in AD patients with controls and in the experimental models of rats and mice which were developed via intracerebroventricular injections of amyloid B oligomers and APP/PS1 in the latter [35]. Amyloid-B oligomers were also found to increase D-serine and serine racemase (SR) levels [35]. However, a 2016 study seems to indicate there is no significant difference in the levels of D-serine in the cerebrospinal fluid of patients affected with AD and other dementias and therefore conclude against the use of CSF d-serine as a biomarker for AD [36]. In contrast to this, Balu et al. found that neurotoxic astrocytes indeed express serine racemase which is the precursor enzyme for the production of D-serine, belying the relational aspect of D-serine to the pathogenesis of AD [37]. A systematic review in 2020 by Chang et al. indicated the increase in levels of D-serine in the serum and CSF of patients affected with AD when compared with controls while another study done in 2020 indicated the unaltered nature of the levels of D-serine in both serum and CSF [38,39]. To complicate matters, D-serine has previously shown to help with recognition and memory in mice, learning and synaptogenesis. A 2021 study done by Piubelli et al. has found that the serum D-serine level and the D-/total serine increased significantly as AD progressed [40]. Taken altogether, we can definitely see a role that D-serine plays in the pathogenesis of AD even though the exact role and mechanism it plays remains to be determined.

The exact role L-serine plays in the pathogenesis of AD is not entirely clear since several studies that has been published appear to contradict one another. In a 2020 paper, Le Douce et al. has indicated that there is an impairment in the glycolysis-derived or de novo synthesis of L-serine in the astrocytes of mice with Alzheimer’s Disease [41]. They went on to show that the cognitive deficits that were present in AD mice were also observed after inactivation of this implicated pathway in hippocampal astrocytes and L-serine supplementation was able to rescue the cognitive deficits displayed in AD mice [41]. Chen et al. 2022 indicated in a letter to the previous paper that their research have shown how there is an increase in phosphoglycerate dehydrogenase (PHGDH) in 6 month old 3xTg AD mice and also in a widespread analysis of the database of postmortem brains of AD afflicted patients [42]. However, the initial group responded by noting that the impairment in the levels of L-serine does not necessarily indicate a decrease in the expression of PHGDH and could be due to the changes in the way glycolysis is affected in early AD but not necessarily due to PHGDH expression. How this expression of PHGDH differs between early and late AD is yet to be explored.

A 2022 study however, showed that D-cycloserine and L-serine were able to rescue some of the neurodegenerative and oxidative effects that were a result of aluminum chloride (AlCl_3_) induced AD in rats [28]. In both the passive avoidance test and the Morris Water Maze test, the group that was treated with either the D-cycloserine or L-serine performed better, with the L-serine group outperforming the former in the passive avoidance test [28]. Neurofibrillary tangles that are a hallmark of AD were also remarkably seen to be attenuated in the treatment groups [28]. The treatment groups also exhibited a decrease in amyloid-beta production through the lower level of expression of genes related to Aβ such as APP [28]. More research needs to be done on other models of AD to further validate the efficacy of L-serine.

## 5. Serines and Schizophrenia

Several studies have demonstrated the relationship between NMDA receptor hypofunction and schizophrenia. Blocking NMDARs in healthy people has elicited symptoms that are akin to those experience by schizophrenic patients. A post-mortem study of 101 healthy controls and 48 patients with schizophrenia were analyzed to have lower levels of the NR1 subunit and NR2C mRNA which could ultimately lead to the alternation of NMDAR stoichiometry and might point to a lower number of NMDAR in schizophrenia [43]. A research study done on serine racemase (SR −/−) knockout mice have shown to exhibit neuroanatomical and neurochemical abnormalities that are seen in schizophrenic patients such as abnormal GABA activity and glutamate signaling [44]. Levels of D-serine have been identified in several studies using the level of *GRIN2A* gene promoter, and found that schizophrenic patients tend to present lower levels of this gene promoter when compared with controls and the D-serine levels also follow the exact same pattern, pointing again to the possibility of D-serine pathway being implicated in the pathogenesis of schizophrenia [45].

Clinical studies have also been performed ever since to uncover the effect of the metabolism of d-serine on schizophrenia. They found that schizophrenia patients had a significantly higher serum levels of DAAO which is the enzyme responsible for the breakdown of d-serine and lower levels of both D-Serine and serine racemace (SR) [46]. A double blind placebo controlled D-serine administration also showed that D-serine at 60 mg/kg/d for 6 weeks provided improvement in the auditory mismatch negativity (MMN) in schizophrenic patients, although it is of note that this specific symptom can have both positive and negative predictive ability in the novel nature of the use of compounds such as d-serine [47]. One of the most recent studies continue to demonstrate the reduced levels of D-serine in the mouse model used to study schizophrenia have adverse effects such as utilizing the “non-ionotropic” NMDA signaling as opposed to the normal ionotropic pathway, leading to instability of the dendritic spines [48]. An early 2023 double-blind, placebo-controlled, clinical trial has also shown that D-serine supplementation of 80 and 100 mg/kg coupled with auditory cognitive remediation (AudRem) engendered an improvement in plasticity in forty-five participants with schizophrenia or schizoaffective disorder [49]. Because L-serine serves as a precursor to D-serine and is relatively innocuous, the effects supplementation of L-serine would have on patients with schizophrenia or schizoaffective disorder should be further investigated, whether it is beneficial in the same way D-serine has been based on the previous studies.

## 6. Serines and Epilepsy

As previously mentioned, astrocytic transmission of D-serine plays an important role in diseases that are NMDA receptor-mediated such as schizophrenia and hypoxia-ischemia [50,51]. Epilepsy particularly status epilepticus (SE) is noted to have prolonged seizure activity that induces hypoxia which becomes severe enough to cause encephalopathy [52]. As such, this form of epilepsy can cause death of neuronal cells, astroglia and epileptogenesis [53,54]. It has also been reported that astroglia death occurred after reactive microgliosis but before damage was inflicted on the neurons in the rat dentate gyrus in pilocarpine-induced SE models [55]. Because D-serine is implicated in glia-to-neuron signaling, it is important to understand the role gliotransmission mediated by D-serine plays in epileptogenesis or seizure activity. A study in 2010 investigated the role D-serine plays in epileptogenesis or seizure activity and deciphered the relationship between the glial responses in the spatiotemporal lobe and the D-serine/serine racemase system within the mesial temporal structures after an SE [56]. Seizure was induced in 9 weeks old male Sprague-Dawley (SD) rats via treatment with lithium chloride and were then given pilocarpine after 20 h [56]. SE is typically observed within 20–30 min after treatment with pilocarpine [56]. The animals are then placed in close observation for about 3 to 4 h daily to check in for behavioral changes and consequent occurrences of seizures for 4 weeks [56]. The brains were then collected. Interestingly, there is a mismatch between D-serine and Serine Racemase Immunoreactivities in the rat hippocampus [56]. Because racemase activity is the main pathway in which D-serine is synthesized under normal conditions, serine racemase immunoreactivity is expected to be colocalized with D-serine. However, the study observed the colocalization with nestin and vimentin instead of D-serine at 1 week after SE [56].

Since SE is not under normal conditions, serine racemase is surmised to function as an eliminase in conditions that are low in ATP [56]. 4 weeks after SE, the immunoreactivity of D-serine was heightened in astrocytes followed by an increase in immunoreactivity of serine racemase [56]. Moreover, in astrocytes, PLK and PNPO immunoreactivities, the PLP synthetic enzymes that act as cofactors in the conversion of L-serine to D-serine, are now also colocalized with SR immunoreactivity [56]. This indicates that D-serine synthesis is increased via serine racemase activations with PLP playing an indirect role in the NMDA receptor-mediated neuronal excitability in rats [56]. Astroglial D-serine Immunoreactivity is also seen to be increased in the rat hippocampus after an SE such that it was higher in the hippocampus proper and hilus 1 week after SE and in the astrocytes in the CA1 region and the dentate gyrus at four weeks after SE, suggesting that astrocytes maybe employing epileptiform discharges through increase in release of D-serine [56]. The study shows how D-serine is implicated in neuronal hyperexcitability and how serine racemase activity might play a part in the way immature astrocytes migrate and differentiate [56].

A study in 2020, however indicated that D-serine might play a role in ameliorating the loss of neuronal cells in temporal lobe epilepsy (TLE) [57]. Pilocarpine was used to induce SE in adult rats and the animals were monitored for seizures and behavioral changes. D-serine was supplied to the animals via mini osmotic pumps and was shown to decrease the number and severity of frank seizures when compared to that of vehicle [57]. The D-serine treatment was also able to rescue the loss of neurons even though the infusion was performed unilaterally into the right hemisphere [57]. Additionally, in order to identify the source of endogenous D-serine in the medial entorhinal area (MEA), the expression of serine racemase (SR) was investigated [57]. Both neurons and astrocytes were positive for serine racemase, indicating that both can be sources of D-serine except for some neurons which could be GABAergic interneurons [57]. In addition to this, the level of D-serine in the epileptic brain is identified through the use of chiral micellar electrokinetic chromatography (MEKC) [57]. Even though total D-serine might be similar between the two conditions (control vs. epileptic), the level of ambient D-serine particularly in the extracellular compartments is deficient in TLE [57].

A subsequent study in 2021 performed immunofluorescence staining and western blotting to identify the expression patters of D-serine and NMDA receptor 1 in patients with intractable epilepsy [58]. The level of D-serine was greater in the neurons and glial cells in patients afflicted with intractable epilepsy than in control participants [58]. The mean absorbance of NMDA receptor 1 was also higher in patients with intractable epilepsy when compared with control participants, indicating the possible implication of this pathway in the Epileptogenesis [58]. The implication of D-serine in Epileptogenesis is certain but how this happens remains to be elucidated. Are the different forms of epilepsy responsible for the varying levels of D-serine observed in the aforementioned studies? Could the levels be attributed to the different timepoints each epileptic event is measured at?

## 7. Serines and Multiple Sclerosis (MS)

Not much is currently known regarding multiple sclerosis since the entire mechanism of MS has yet to be fully elucidated. However, due to the nature of MS being an inflammatory disease and the immune system and its derived cells requiring constant nutrients to maintain their functions, amino acid pathways that contribute to this homeostatic functioning could be implicated in mitigating the symptoms of the disease. Serine/threonine kinases have been demonstrated to be utilized by cells to sense the levels of amino acids particularly the GCN2K and the mammalian target of rapamycin (mTOR) pathway [59]. A lower level of amino acid will cause a deacetylation of tRNAs and these tRNAs will activate GCN2K [60]. The kinase will then phosphorylate Ser 51 of eukaryotic initiation factor 2 (eIF2α)-associated 67-kDa glycoprotein which is required to initiate translation in eukaryotes [61]. Even though the direct effects of L-serine in MS are yet to be fully known, it is an area that has room for great exploration since amino acid homeostasis is important in the process of inflammation.

## 8. Clinical Studies on L-serine and Neurological Diseases

There have not been widespread clinical studies done on L-serine since most if not all have focused directly on D-serine. However, recent studies have shown that d-serine might have a longer time to diffuse through the blood-brain barrier and might even engender nephrotoxicity [41,62]. Worse yet, long term effects of d-serine are still not entirely known. In contrast to this, L-serine has been approved for use by the FDA [9,63]. Several clinical studies however has been done on L-serine, the first of which was on Hereditary sensory neuropathy type 1 (HSAN1). L-serine supplementation of 400 mg/kg/day for 52 weeks was able to reduce the neurotoxic level of 1-deoxysphingolipds which is responsible for the degeneration without causing other metabolic side effects [64]. A phase I clinical trial was also performed on Amyotrophic Lateral Sclerosis (ALS) patients to uncover the efficacy of oral L-serine supplementation [65]. They were able to find that L-serine did not pose a threat to the patients and did not appear to contribute to the rate of decline. A phase II trail is now planned to investigate the effects [65]. The aforementioned *GRIN2B* gene autosomal dominant mutations have led to severe encephalopathy and was seen in a 5 year old patient who had severe encephalopathy. The GluN2B-containing NMDARs were found to have a smaller pore size through structural modeling and thorough expression on cellular models appeared to have lower glutamate affinity [65]. Because the naturally occurring d-serine tend to restore the levels of NMDAR activity, L-serine was started on the patient and the patient exhibited improvement in motor and cognitive performance and even communication after 11 and 17 month of supplementation [65].

A similar clinical study was performed on 2 female patients, one 18 months old and the other 4 years old to investigate the effects of L-serine supplementation (500 mg/kg/day in 4 doses) in *GRIN2B*-related neurodevelopmental disorder that arises out of a loss of function of *GRIN2B* gene [66]. The GluN2B-containing NMDARs no longer functions well due to this. One patient was seen to exhibit improvements in psychomotor development and caregivers report noticing increased alertness and better communication skills [66].

However, more studies need to be performed before we can safely apply the usage of L-serine for the wide variety of neurological diseases that we have outlined above. L-serine due to its relatively safe-to-use nature left us with an open field of utilization for all applications. A summary of the current research regarding serines and neurological and neurodegenerative disorders are displayed in Table 1.

## 9. Conclusions and Future Directions

Literature up until this point has been concentrated on the study of D-Serine but because of its toxic nature and underreported long-term effects, L-serine might be a useful substitute as it is a precursor to D-Serine. However, it is important to also understand the levels of serine racemase (SR) in the neurological diseases that we listed above because the enzyme serine racemase is needed to convert L-serine to D-serine and if the level of SR is already low in patients which it was in a study shown above, L-serine use might be to no avail. The pathway remains an area that could be used to target a wide range of neurological and neurodegenerative diseases but it is crucial to pay attention to the ages of the animals we conduct our experiments on since diseases such as Alzheimer’s are age-related and in some ways Parkinson’s. The levels of these amino acids and enzymes could differ vastly among the age ranges and lead us to conclude without much basis. Even though this may be the case, the purported benefits of L-serine due to its position in the pathway allows for a wide field of usage and potentially be employed to elicit a great deal of beneficiary effects. As such, a greater but carefully measured emphasis should be placed on uncovering the exact ways in which L-serine work particularly in the pathogenesis of certain neurological and neurodegenerative diseases and the baseline levels of the different agents, both enzymes (such as PHGDH and Serine Racemase) and substrate s(both L-serine and D-serine) that are involved in the pathway at different timepoints.

## Figures and Tables

**Table 1:** Overview of the Serine-related research studies.

Disease	Area of Research	Effects	References
Parkinson’s Disease (PD)	D-serine	D-serine rescued/mitigated some behavioral and motor deficits caused by PD	[32]
Alzheimer’s Disease (AD)	D-serine levels	Conflicting	[37,38,39]
	L-serine	Conflicting (PHGDH vs. L-serine)	[41,42]
Epilepsy	Astroglial D-serine immunoreactivity	Increase in the level of D-serine via epileptiform discharges in astrocytes	[56]
	D-serine’s role in temporal lobe epilepsy (TLE)	Mitigated loss of neurons, ambient D-serine depleted in TLE, and similar total D-serine	[57]
	Expression patterns of D-serine and NMDA receptor 1 in patients with intractable epilepsy	Both levels higher in patients with intractable epilepsy	[58]
Multiple Sclerosis	Amino acid homeostasis.	N/A	-
Schizophrenia	Serine racemace (SR) knockout.	Exhibit neuroanatomical and neurochemical abnormalities that are seen in schizophrenic patients	[44]
	D-serine at 60 mg/kg/d for 6 weeks.	Improvement in auditory mismatch negativity	[47]
Amyotrophic Lateral Sclerosis (ALS), Hereditary Sensory and Autonomic Neuropathy (HSAN) Type 1C, and Severe Encephalopathy	L-serine administered to patients with severe encephalopathy	Improvement in motor and cognitive performance and even communication after 11 and 17 months of supplementation	[62]
	L-serine supplementation of 400 mg/kg/day for 52 weeks	Reduced the neurotoxic level of 1-deoxysphingolipds	[64]
	L-serine phase I trial on ALS patients	Did not appear to contribute to the rate of decline, so phase II was started	[65]

## Data Availability

Not Applicable.

## Abbreviations

AD	Alzheimer’s Disease
ALS	Amyotrophic Lateral Sclerosis
CSF	Cerebrospinal Fluid
DAAO	D-amino acid oxidase
EEG	Electroencephalography
GCN2K	General Control Nonderepressible2 Kinase
GRIN2B	Glutamate(NMDA) Receptor Subunit Epsilon-2
HSAN1	Hereditary sensory neuropathy type 1
MMN	Mismatch Negativity
MPTP	(1-methyl-4-phenyl-1,2,3,6-tetrahydropyridine)
mTOR	Mammalian Target of Rapamycin
mTORC1	Mammalian Target of Rapamycin Complex 1
NK Cells	Natural Killer Cells
NMDAR	N-methyl D-aspartate receptor
PD	Parkinson’s Disease
PHGDH	Phosophoglycerate Dehydrogenase
PLP	Pyridoxal Phosphate
PPAR-y	Peroxisome Proliferator-activated Receptor Gamma
PSAT	Phosphohydroxythreonine Aminotransferase
PSP	Phosphoserine Phosphatase
SE	Status Epilepticus
SR	Serine Racemase
TH+	Tyrosine Hydroxylase-positive
